# Serum Inhibin A and Inhibin B Levels in Epithelial Ovarian Cancer Patients

**DOI:** 10.1371/journal.pone.0090575

**Published:** 2014-03-05

**Authors:** Pawel Walentowicz, Magdalena Krintus, Pawel Sadlecki, Marek Grabiec, Aneta Mankowska-Cyl, Alina Sokup, Malgorzata Walentowicz-Sadlecka

**Affiliations:** 1 Department of Obstetrics and Gynecology, L.Rydygier Collegium Medicum in Bydgoszcz, Nicolaus Copernicus University, Bydgoszcz, Poland; 2 Department of Laboratory Medicine, L.Rydygier Collegium Medicum in Bydgoszcz, Nicolaus Copernicus University, Bydgoszcz, Poland; 3 Department of Gastroenterology, Angiology and Internal Diseases, Nicolaus Copernicus University, Dr. J. Biziel University Hospital, Bydgoszcz, Poland; University of Nebraska Medical Center, United States of America

## Abstract

**The aim:**

of our study was to examine serum inhibin A and inhibin B concentrations in ovarian cancer patients in relation to clinicopathological features and 5-year survival.

**Material and Methods:**

We enrolled 90 epithelial ovarian cancer patients in our study, aged 45–81 years, who underwent optimal cytoreductive surgery. In all patients, serum inhibin A and inhibin B concentrations were measured using a two-step sandwich type enzyme immunoassay before surgery.

**Results:**

In the group of patients with ovarian cancer median serum concentration of inhibin A was 3.87 pg/mL (0.96–10.09) and inhibin B was 13.9 pg/mL (5.1–45.0). Median concentrations of inhibin A and B in relation to FIGO stage and histological subtype did not differ significantly. Inhibin A levels were significantly higher in patients with lower grading (G1 and G2) in comparison to those with higher grade G3 (p = 0.001). There were no differences in inhibin B concentrations in relation to grading. The Kaplan-Meier analyses demonstrated no differences in survival rate in relation to inhibin A levels, while there was a stepwise impairment of 5-years survival with increased inhibin B level. In the group of patients with inhibin B levels higher than 20 pg/ml the survival rate was lower (p = 0,00625, log-rank test).

**Conclusion:**

1. Higher inhibin A serum levels were found in patients with highly differentiated ovarian carcinoma compared to the group of patients with a poorly differentiated cancer, which may confirm the influence of inhibin A on cell proliferation processes. 2. A significant importance of inhibin B was demonstrated in the prediction of death within less than a five year period. The probability of survival in patients featuring high inhibin B levels was lower with statistical significance. This may indicate the need for further studies on how to block the inhibin B activation pathway in the ovarian carcinoma therapy.

## Introduction

Epithelial ovarian cancer is the fifth leading cause of death in women in Europe and the United States [1]. Although understanding of ovarian cancer has improved a lot, the etiology and the course of the disease remain unknown [2]. The conditions that enable the cancer cells to initiate metastases to distant sites represent a crucial but still poorly understood clinical issue. Moreover, even though new markers were introduced to the clinical practice, more than 70% of patients present with advanced disease (stage III or IV), and long-term survival rates are low (around 30% only) [3], [4], [5].

Inhibins were originally isolated from gonadal fluids based on their respective abilities to inhibit follicle stimulating hormone (FSH) secretion from the pituitary. Subsequently, these proteins were identified as members of a family of growth factors, the transforming growth factor-beta (TGFβ) superfamily, with multiple functions as local regulators of gonadal biology [6], [7]. Inhibin A and inhibin B belong to a small sub-group of the TGF-β superfamily that act as antagonists and are structural homologues of activins, comprising the activin β-subunit and a unique α-subunit. Heterodimerisation gives rise to inhibin A (α/βA) and B (α/βB) [8], [9]. In its structure, the mature inhibin A dimer is similar to activin A [10].

Inhibins block the actions of activin at the pituitary [11]. Sharing the β-subunit, inhibin can bind activin type II receptors, with a lower affinity [12]. A membrane bound proteoglycan, betaglycan, acts as an inhibin co-receptor and moreover betaglycan has been characterised as an accessory receptor for related TGF-β ligands, TGF-β1, -β2 and -β3 [13]. In the presence of betaglycan, inhibin forms a high affinity complex with the activin type II receptor [13]. This interaction inhibits activin's access to its type II receptors, and blocks the intracellular signaling cascade [14].

The fact that inhibins expression is restricted to ovarian granulosa cells in women, led to establish inhibin levels as a useful serum marker of granulosa cell tumours (GTCs).

Moreover as inhibin A and B belong to transforming growth factors family and their representatives play an important role in ovarian cancer carcinogenesis, it can be hypothesized that they could also play a role in ovarian carcinogenesis. It was shown that some regions of genes encoding inhibins tend to be impaired in ovarian carcinomas, however a relationship between these damages and carcinogenesis processes in epithelial ovarian cancer patients has not been elucidated [15]. One of suggested mechanisms participating in the carcinogenesis of ovarian carcinoma is the activin pathway disturbances resulted from the decreased expression of β-glycans. Similarly to other neoplasms, ovarian cancer is characterized by the loss of β-glycan expression, yet the role of activin pathway, and interactions among inhibins, activins and other components of TGF-β family, still remain unclear [15].

Considering those facts the aim of this study was to examine inhibin A and inhibin B concentration in the serum of epithelial ovarian cancer patients in relation to clinicopathological features, such as a pathological subtype of the tumor, FIGO stage, grading, menopause status and overall 5-year survival.

## Patients

We enrolled 90 epithelial ovarian cancer patients aged 47–81 years (mean 59.6+/− 10.3 years) treated in Department of Gynecology and Obstetrics, Ludwik Rydygier Collegium Medicum in Bydgoszcz in the period of 2005–2006. All women were postmenopausal.

Only women who underwent optimal cytoreductive surgery were considered for further analysis, all of them with residual cancerous focuses smaller than 1 cm in diameter. All patients were operated by experienced gynecological oncologist. The standard surgical protocol included tumorectomy, hysterectomy with bilateral salpingoovariectomy, pelvic lymphadenectomy, omentectomy and appendectomy. International Federation of Gynecology and Obstetrics (FIGO) ovarian cancer staging system was used to assess clinical stage of the disease. Early stages were confirmed in 26 patients (FIGO I in 16 women, II in 10) and advanced disease in 64 patients (FIGO III–56, IV–8). All ovarian cancer cases were with histological confirmation, of which 68 (75%) serous, 7 (8%) mucinous and15 (17%) endometrioid. Histological examination was performed at the Pathology Department, University Hospital in Bydgoszcz, and histological grading was determined (G1 in 13, G2 in 28 and G3 in 49 patients).

After optimal cytoreductive surgery, all women underwent 6 courses of chemotherapy based on carboplatin and paclitaxel. Baseline characteristics of the study participants are shown in Table 1.

**Table 1:** Baseline characteristics of the study group.

Parameter	Patients with ovarian cancer (n = 90)
Age (years)	59.6±10.3
FIGO stage n (%)	
I	16 (17.8%)
II	10 (11.1%)
III	56 (62.2%)
IV	8 (8.9%)
Histological subtype n (%)	
Serosum	68 (75%)
Mucinous	7 (8%)
Endometrioid	15 (17%)
Histological grading n (%)	
G1	13 (14.4%)
G2	28 (31.1%)
G3	49 (54.4%)

The Bioethical Committee at the Ludwik Rydygier Collegium Medicum, Nicolaus Copernicus University in Torun have reviewed and approved this study. All participants have provided written, informed consent.

## Methods

Blood samples were collected (10 ml) after admission to the hospital, on the day before surgery. After centrifugation in standard conditions serum was obtained, aliquoted and stored at −70°C until assayed.

Serum inhibin A and inhibin B concentrations were measured using an enzymatically amplified two-site two-step sandwich-type immunoassay (Inhibin-A/B ELISA; Diagnostic Systems Laboratories, Inc., Webster, Texas, USA). The imprecision of the assay for inhibin A was 6.2% at 23 pg/mL and 3.0% at 781 pg/mL. The lowest inhibin A concentrations in a sample which could be detected was 1 pg/mL (lower detection limit). As a reference value for women over 45 years we accepted inhibin A concentration of 1–3.88 pg/mL.

Intra- and inter-assay coefficients of variation with serum controls for inhibin B were approximately 3.5%–5.6% and 6.2%–7.6%, respectively. The lower limit of detection was 6 pg/mL. Assay measured range for inhibin B was 10–531 pg/mL.

The Kolmogorov-Smirnov test was used to assess normality of distribution of investigated parameters. Data were expressed as mean ± standard deviation and median with 25th–75th percentiles. Comparison between the groups was performed by using the Mann-Whitney test and the Kruskal-Wallis test for non-Gaussian distributed variables with the post hoc Dunn's tests. P value<0.05 was considered statistically significant.

Overall survival rate was examined for significance using log-rank test and Kaplan-Meier curves.

Univariate and multivariate Cox regressions were performed. For the analysis, a forward selection with a P value of less than 0.05 for entry was applied. The effects of the variables were expressed as hazard ratios per 1 SD change to allow for a better comparability between the effect sizes of the different tested variables.

All statistical analyses were performed using Statistica 10.0 for Windows (StatSoft, Tulsa, OK, USA).

## Results

In the group of patients with ovarian cancer median serum concentration of inhibin A was 3.87 pg/mL (0.96–10.09) and inhibin B was 13.9 pg/mL (5.1–45.0).

Median concentrations of serum inhibin A and B in relation to FIGO stage did not differ significantly. However, there was a trend to statistical significance for inhibin A (Table 2).

**Table 2:** Inhibin A and Inhibin B concentrations in relation to FIGO stage.

Variable	FIGO Stage	Q1	Q2	Q3	U Mann-Whitney Test P value
Inhibin A (pg/mL)	I/II	4.11	7.65	13.9	0.07
	III/IV	0.86	2.49	7.18	
Inhibin B (pg/mL)	I/II	5.11	7.61	27.88	0.44
	III/IV	5.41	15.82	44.2	

FIGO stage- the International Federation of Gynecology and Obstetrics stage; Q1: lower quartile; Q2: median; Q3: upper quartile.

Median serum inhibin A levels were significantly higher in patients with lower grading (G1), as well as in patients with G2 in comparison to those with higher grade G3 (p = 0.001). Considering serum inhibin B concentrations in relation to grading, there were no differences observed (Table 3).

**Table 3:** Inhibin A and Inhibin B and concentrations in relation to grading.

Variable	Grading	Q1	Q2	Q3	Kruskal-Wallis test p value
Inhibin A (pg/mL)	1	6.89	8.23	11.61	0.001
	2	2.49	6.8	18	
	3	0.57	0.96	2.87	
Inhibin B (pg/mL)	1	1.76	3.93	6.97	0.12
	2	6.34	10.24	32.7	
	3	6.03	22.47	46.3	

Q1: lower quartile; Q2: median; Q3: upper quartile.

No significant differences were found between inhibin A and B concentrations and histopathological subtype of the tumor (Table 4).

**Table 4:** Inhibin A and inhibin B concentrations in relation to the histological type of cancer.

Variable	Histological Type	Q1	Q2	Q3	U Mann-Whitney Test P value
Inhibin A (pg/mL)	Non-serous	0.00	7.08	12.81	0.498
	Serous	0.19	2.87	7.47	
Inhibin B (pg/mL)	Non-serous	5.88	6.97	27.88	0.961
	Serous	5.11	17.65	43.12	

Q1: lower quartile; Q2: median; Q3: upper quartile.

**Figure 1:**
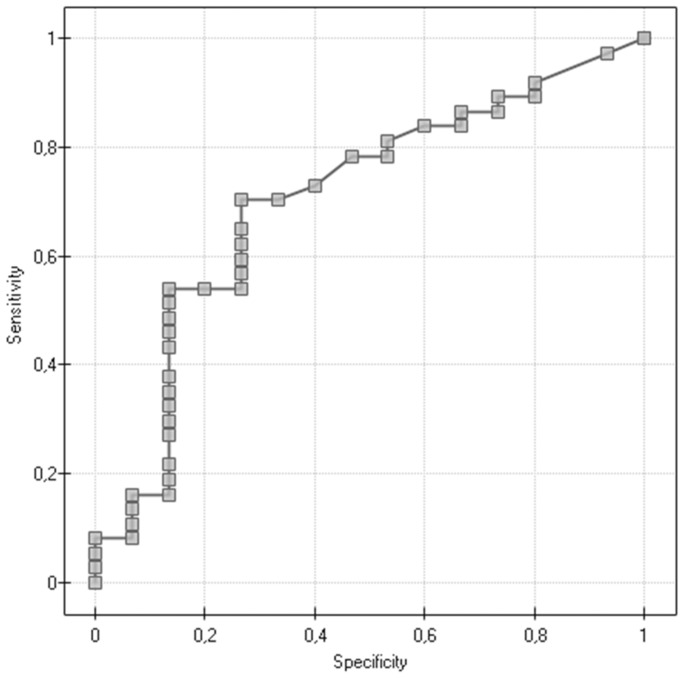
Prediction of death within five years for the variable inhibin B (pg/mL).

We evaluated the ROC curves to assess diagnostic accuracies of investigated variables for the prediction of death within five years (Table 5, Fig. 1). The highest level of discrimination was found for inhibin B (area under the curve [AUC] = 0.705), however, this was not significantly different from the diagnostic accuracy of two other variables: age (AUC = 0.629), and inhibin A (AUC = 0.640).

**Table 5:** The diagnostic value of age and inhibin A and B for the prediction of death within five years.

Variable	AUC	SE	−95% CI	+95% CI
Age	0.629	0.117	0.414	0.811
Inhibin A (pg/mL)	0.640	0.124	0.425	0.820
Inhibin B (pg/mL)	0.705	0.109	0.480	0.861

Moreover, the association between inhibin A, Inhibin B and survival rate was also performed according to univariable and multivariable Cox regression analysis (including variables such as: age, histological subtype (serous), high grade (G2+G3), advanced FIGO stage (Table 6, 7). Grade and advanced FIGO stage were statistically important prognostic factors in univariate analysis for overall survival (Table 6). In multivariate analysis only advanced FIGO stage was statistically important (Table 7).

**Table 6:** Prognostic factors for overall survival selected by Cox's univariate analysis.

	Parameter evaluation	p–value	HR	HR (95%Cl)	
				−95% CI	+95% CI
Age	0.014008	0.055647	1.027172	0.999353	1.055764
Inhibin A (pg/mL)	0.000068	0.975167	0.999998	0.999864	1.000132
Inhibin B (pg/mL)	0.000269	0.349948	1.000251	0.999725	1.000778
Hist-Pat (serosum)	0.184654	0.790873	1.102888	0.534780	2.274509
Grading (2+3)	0.505748	0.039438	0.124523	0.017150	0.904140
FIGO III/IV	0.262220	0.001529	0.189755	0.067887	0.530392

Cl: confidence interval; FIGO: Federation Internationale de Gynecologie et d'Obstetrique; HR: hazard ratio.

**Table 7:** Prognostic factors for overall survival selected by Cox's multivariate analysis.

	Parameter evaluation	p–value	HR	HR (95%Cl)	
				−95% CI	+95% CI
Age	0.020309	0.380937	1.017953	0.978230	1.059289
Inhibin A (pg/mL)	0.000083	0.328069	0.999919	0.999757	1.000081
Inhibin B (pg/mL)	0.000284	0.345725	1.000268	0.999711	1.000825
Hist-Pat (serosum)	0.210098	0.705970	1.171786	0.514253	2.670052
Grading (2+3)	0.523662	0.196379	0.258444	0.033180	2.013027
FIGO (III+IV)	0.275151	0.008129	0.233057	0.079258	0.685298

Cl: confidence interval; FIGO: Federation Internationale de Gynecologie et d'Obstetrique; HR: hazard ratio.

**Figure 2:**
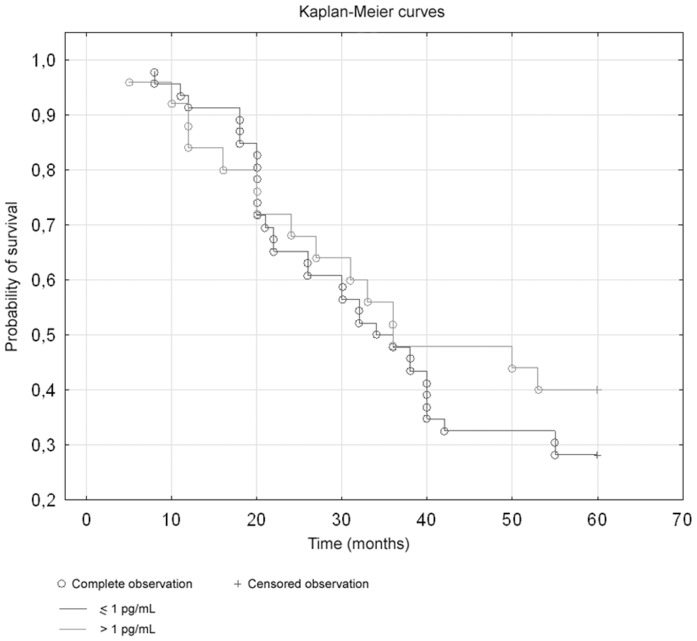
Survival curves in relation to inhibin A levels (p = 0,46, log-rank test).

**Figure 3:**
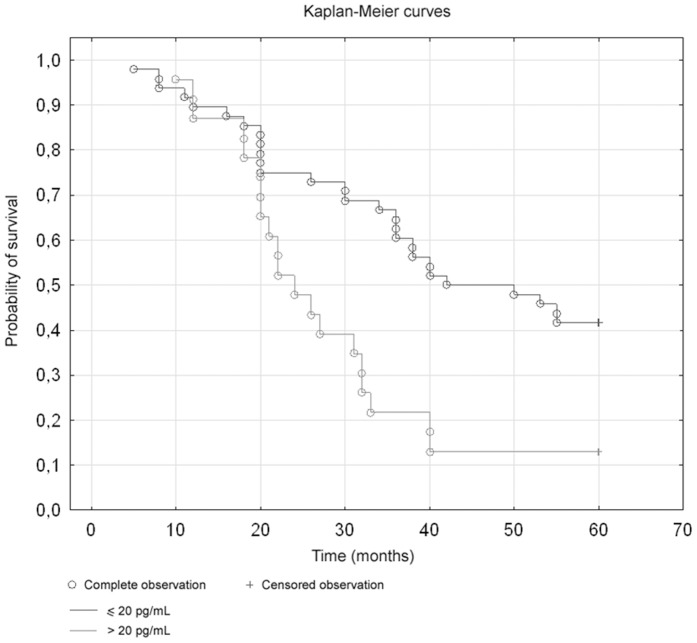
Survival curves in relation to inhibin B levels (p = 0,00625, log-rank test).

Moreover, the Kaplan-Meier analyses were performed. There were no differences in survival rate in relation to inhibin A levels (Figure 2). The Kaplan-Meier analyses demonstrated a stepwise impairment of cancer 5-year survival with increased inhibin B level. The Log-rank test showed significant differences between inhibin B concentration and survival; in the group of patients with inhibin B levels higher than 20 pg/ml the survival rate was lower (p = 0,00625, log-rank test) (Figure 3).

## Discussion

Ovarian cancer still remains a substantial problem of the present oncologic gynaecology associated with a late diagnosis, a low treatment efficacy and a high mortality. New methods of the ovarian carcinoma diagnostics are introduced on a regular basis, however such actions do not significantly improve treatment outcome [16]. It is of great importance to search for novel factors that would enable us to prognosticate treatment outcomes and to help us select a group of female patients with the poor prognosis who require a more aggressive therapy.

In our study, we have evaluated serum levels of hormones belonging to TGF-β family, namely inhibin A and inhibin B. We found that A and B inhibin concentrations did not significantly differ depending on the level of clinical development (FIGO stage) and the type of histopathological examination. Inhibin A levels did not correlate with a 5-year survival rate. Due to our knowledge we are the first to have evaluated inhibin A levels with regards to 5-year survival rate in women with epithelial ovarian carcinoma. There are some reports in the literature on inhibin A level in patients affected by epithelial ovarian carcinoma. In their study, Roberts et al. demonstrated blood plasma elevated concentrations of inhibin alpha-subunit in ovarian carcinoma patients [17]. The authors did not find any relationship between inhibin A and clinicopathological characteristics of the tumour, which is in line with our own studies.

Nevertheless, we have demonstrated a positive correlation between increasing inhibin A levels and the level of histological differentiation. In the subgroup of patients, in whom the tumour was characterized by a low grade (G1), the highest inhibin A levels were noted (8.23 pg/mL for G1 vs. 0.96 pg/mL for G3, p = 0.001).

Knight and Glister proved that the balance between inhibins and activins, produced by granular cells, is essential in the regulation of numerous factors connected with follicular development including cell proliferation [18]. Perhaps, if an imbalance in such a system occurs, it would affect an enhanced proliferation of ovarian carcinoma cells.

Based on our study results, we have found differences in inhibin A levels among women affected by the ovarian carcinoma, whereas there was no difference of inhibin B levels among them. Perhaps, this phenomenon is – to some extent – related with tumour cell proliferation disorder in ovarian carcinoma patients. We consider it needs to be confirmed by further studies also including the evaluation of activin levels.

Our studies are pioneering works on the role of inhibin A in the epithelial ovarian carcinoma. For the first time, we were unlucky to confirm the purposefulness of inhibin A level routine evaluation in the prediction of clinicopathological characteristics in patients affected by the epithelial ovarian cancer.

We have also studied the levels of inhibin B, another hormone belonging to TGF-β family. Inhibin B levels did not reveal statistically significant differences depending on the degree of clinical development, the level of histological malignancy and the histological type of cancer. However, using ROCs (Receiver Operating Curves), we have found a correlation between a preoperative blood plasma inhibin B level and a 5-year survival time. By carrying out a survival time analysis with the help of Kaplan-Meier curves, we have observed a significantly shorter time to death in the group of patients with inhibin B levels over 20 pg/mL.

Due to our knowledge we were the first to have analyzed an association between inhibin B levels and patients' survival time. In the available literature, we have not found reports on the inhibin B effect on the ovarian carcinoma patients' survival time. We think that the increase of inhibin B level may be presumably due to the blockage in the transduction signal pathway of activin. Some authors suggest that the activin signal pathway exerts an inhibitory effect on tumour growth, quite similar to TGF-β action in normal epithelial cells. The weakening of activin activity may lead to the loss in the enzyme inhibitory ability induced onto growth, and inhibin B overproduction. A reduced β-glycan expression may be a possible intermediary mechanism involved in this process, however interactions taking part among respective agents of TGF-β family are unusually complex [19]. Both inhibin A and inhibin B bind β-glycan solely at the binding site located in the proximal region of the cell membrane [20], [21]. It has been revealed that the binding sites are common for inhibins and other components of TGF-β family, yet they differ themselves among one another with regard to specific amino acid residues characteristic for respective factors [20]. It seems to be probable that inhibin A and inhibin B present a different affinity for β-glycan [22]. Inhibin A binds with a higher affinity for β-glycan and type II receptor in contrast to inhibin B [23]. Nevertheless, inhibin B is characterized by a greater ability to antagonize FSH release from the pituitary gland, which is indicative of possible inhibin B binding to activin type II receptor via another pathway [22].

We postulate that activin mediated growth arrest accompanied by a consequent elevation of inhibin concentrations may possibly constitute an important stage in the carcinogenesis of the ovarian carcinoma.

Inhibin A, belonging to TGF family, has an impact on NF-κB (Nuclear Factor kappa-light-chain-enhancer of activated B cells) signal pathway. NF-κB is a protein complex acting as a transcription factor. NF-κB signal pathway activation occurs in ovarian carcinoma tumours of epithelial origin [24]. Initiation of this process may be not only due to a mutation but also to the presence of inflammatory process inducers in the tumour microenvironment. As a result of NF-κB pathway activation, target genes responsible for an enhanced proliferation, infiltration, metastasizing, and angiogenesis get activated [25]. The above mentioned processes determine an aggressive phenotype of the tumour [26], [27].

The components of TGF-β superfamily frequently exert a synergistic action with FSH, therefore it is crucial to consider the fact that a number of signal transduction pathways may be activated simultaneously. It has been found that activin, via Smad2/3 receptors, activates PI3 Akt pathway, existing in epithelial ovarian carcinomas, and affects the overexpression of antiapoptotic genes [28], [29]. Complicated interactions among inhibin, activin, estrogens and NF-κB signal pathway have been proven [30]. It is commonly known that TGF-β family factors and estrogens play a pivotal part in ovarian functions, however NF-κB role still remains obscure [31], [32], [33]. On the other hand, it is probable that inhibin, activin, estrogens and NF-κB play some role in the pathogenesis of the ovarian carcinoma. Mechanisms underlying this process cover the impairment of proliferation and apoptosis. Each of the signal transduction pathways described above contains mutual factors. Hence, their cross-interactions are possible.

Epithelial ovarian cancer remains one of the most aggressive disease, particularly in women of a high socioeconomic status who live in industrialized countries. Over the recent years, along with an intensive development of molecular biology, numerous studies on the mechanism of the ovarian carcinogenesis have been published [34], [35]. In spite of that, the disease is often diagnosed after tumour cells have disseminated within the peritoneal cavity, at the late stages of clinical development, and the percentage of successfully treated patients has remained almost at an unchanged level for several decades. Epithelial ovarian cancer is an aggressive disease for which there are few effective biomarkers and therapies [36].

Importance of inhibin A and inhibin B in epithelial ovarian cancer is relevant. Alteration of the inhibin/activin pathway may contribute to the development of epithelial ovarian cancer due to the alteration of the crosstalk between granulosa and epithelial cells. In their recent studies Tournier at al. identified a single de novo mutation (c.1157A>G/p.Asn386Ser) within the INHBA gene encoding the βA-subunit of inhibins/activins, which play a key role in ovarian development [36]. Furthermore, in a cohort of 62 cases, they detected an additional unreported germline mutation of the INHBA gene (c.839G>A/p.Gly280Glu). Authors provide arguments indicating that germline inhibin mutations contribute to the genetic determinism of epithelial ovarian tumors by altering the inhibin/activin production. Results obtained by authors strongly suggested that inhibin mutations contributed to the genetic determinism of epithelial ovarian tumors [37]. The impact of the INHBA mutation on inhibin/activin production and the role of the inhibin pathway in ovaries and ovarian carcinogenesis is of great interest.In conclusion, based on our studies, we have observed shorter 5-years survival rate in the group of patients with inhibin B levels over the upper normal limit. Perhaps, the inhibin B evaluation in clinical practice might help find a group of epithelial ovarian cancer patients, in whom prognoses are bad enough to use more aggressive treatment methods. This presumption require further studies including the ones on the possibilities to block inhibin B activation pathways in the ovarian carcinoma therapy. One should also pay attention to the fact that in the studied group of patients merely the level of clinical development, according to FIGO, was an independent predictor of 5-year survival rates based on a multivariable analysis, which highlights an essential role of properly performed surgical staging.

Hopefully, studies conducted by us will lead to the development of further diagnostic assays allowing for a more accurate prediction of cancer treatment outcomes.

## Conclusions

1. Higher inhibin A serum levels were found in patients with highly differentiated ovarian carcinoma compared to the group of patients with a poorly differentiated cancer, which may confirm the influence of inhibin A on cell proliferation processes.

2. A significant importance of inhibin B was demonstrated in the prediction of death within less than a five year period. The probability of survival in patients featuring high inhibin B levels was lower with statistical significance. This may indicate the need for further studies on how to block the inhibin B activation pathway in the ovarian carcinoma therapy.
